# The Information Coded in the Yeast Response Elements Accounts for Most of the Topological Properties of Its Transcriptional Regulation Network

**DOI:** 10.1371/journal.pone.0000501

**Published:** 2007-06-06

**Authors:** Duygu Balcan, Alkan Kabakçıoğlu, Muhittin Mungan, Ayşe Erzan

**Affiliations:** 1 Department of Physics, Faculty of Sciences and Letters, Istanbul Technical University, Istanbul, Turkey; 2 Department of Physics, Faculty of Arts and Sciences, Koç University, Istanbul, Turkey; 3 Department of Physics, Faculty of Arts and Sciences, Boğaziçi University, Istanbul, Turkey; 4 Gürsey Institute, Istanbul, Turkey; IBM Thomas J. Watson Research Center, United States of America

## Abstract

The regulation of gene expression in a cell relies to a major extent on transcription factors, proteins which recognize and bind the DNA at specific binding sites (response elements) within promoter regions associated with each gene. We present an information theoretic approach to modeling transcriptional regulatory networks, in terms of a simple “sequence-matching” rule and the statistics of the occurrence of binding sequences of given specificity in random promoter regions. The crucial biological input is the distribution of the amount of information coded in these cognate response elements and the length distribution of the promoter regions. We provide an analysis of the transcriptional regulatory network of yeast *Saccharomyces cerevisiae*, which we extract from the available databases, with respect to the degree distributions, clustering coefficient, degree correlations, rich-club coefficient and the *k*-core structure. We find that these topological features are in remarkable agreement with those predicted by our model, on the basis of the amount of information coded in the interaction between the transcription factors and response elements.

## Introduction

With the development of high throughput experimental techniques [1] a large amount of data on gene interactions [2] is now available [3]–[6], revealing a complex network. The organizational principles underlying these genetic regulatory networks are of great experimental [3], and theoretical [10]–[15] interest.

In this paper we would like to present an information theoretic approach to modeling genetic interaction networks. We believe that this approach provides an understanding of how interactions based on shared information might arise spontaneously between subsequences of any sufficiently long linear code, even when this code is completely random, and how a complex network emerges as a result.

We construct a null model of a transcriptional regulatory network (TRN) by adapting the “sequence-matching” rule which we have introduced earlier [16], [17] as a condition for the establishment of edges between nodes of a network. A sequence S is said to match another sequence S′ if it is contained in S′ as an uninterrupted subsequence. In the case of the TRN, the nodes consist of genes, with their associated promoter regions (PRs) and the transcription factors (TFs) which they code, if any. To model this network, we label TFs by the binding sequences they recognize, and represent the PRs by another set of (typically longer) sequences. The conditions that need to be satisfied for a TF to recognize and bind a specific DNA sequence within a promoter region are mimicked by the sequence-matching rule between the sequences associated with the respective TFs and PRs.

The biological input to the model consists of the effective length distribution of the binding sequences recognized by the transcription factors of yeast [3], [9] and the form of the length distribution of the intergenic regions [18], in the absence of more specific data regarding the lengths of the promoter regions. By effective length we mean the bit-wise information content (site specificity) of a binding motif together with its variations and experimental uncertainties in its determination. The empirical binding sequences of the TFs are reported [3], [9] in an extended alphabet (e.g. “rACGCGt” for the transcription factor MBP1) specifying the base preferences or the binding affinities as a set of letters with variable case. We have converted the information contents coded via this extended alphabet into to a binary code by assigning zero, one and two bits representing low, medium and high information contents (see the Results and the Methods sections).

Our model is a null model in the sense that no further knowledge specific to the TRN of yeast is provided apart from the distribution of the amount of information coded in the cognate response elements; no assumptions are made regarding the actual amino acid code of a TF, nor its possible three dimensional folding pattern; all possible refinements involving steric constraints, chromatin folding, trans-vs. cis-regulation, etc. have been neglected. A TF is simply labeled by a binary string whose length is drawn from the effective bit-length distribution of the cognate response elements, and this string is then queried in the randomly composed strings representing the PRs, to establish an interaction. Since the specific content of the strings does not enter into the assumptions of the model, the strings are composed randomly, with uniform probabilities. Thus our null model is designed to test the null hypothesis that the topology of the TRN is essentially determined by the exchange of information between the cognate/cognate response elements, of given length distributions.

In order to test the predictions of this model against real data, we make a detailed analysis of the topological features of the directed network corresponding to the TRN of yeast using the available data [3]–[6] (see Table 1 for the databases used). We have also investigated (supporting Text S1) the frequency of “3-motifs,” namely the frequency of occurrence of different directed edge configurations which may be found within connected sub-graphs containing 3 nodes.

**Table 1:** Yeast databases.

Source	Genes	TFs	Interacting Pairs
Fraenkel Lab^a^ [3]	2884	102	6441
Luscombe et al.^b^ [4]	3459	142	7071
Yeastract^c^ [5]	4252	146	12530
Kınıkoğlu et al.^d^ [6]	3763	180	9135
Model	4167±177	202±14	1436 5±2067

a http://fraenkel.mit.edu/Harbison/release_v24/bound_by_factor/

b http://sandy.topnet.gersteinlab.org/index2.html

c http://www.yeastract.com

d *private communication*

The number of interacting genes, TFs, and interacting pairs that appear in the yeast regulatory network as obtained from different sources [3]–[6], and the average values, obtained from one hundred realizations of our model (±the standard deviations) with *μ* = 0.1.

We demonstrate that our model is able to capture with convincing precision all the global topological features of this directed network such as the distribution of the in-, out- and total degree, i.e., number of neighbors per node [19]–[22], the clustering coefficient [19]–[22] measuring the probability that the neighbors of a node are connected to each other, the degree-degree correlation [23], [24], namely the correlation between the respective number of neighbors of neighboring nodes, the rich-club coefficient [25], [26] indicating the extent of clustering among highly connected nodes, and the *k*-core structure [19], displaying the hierarchical organization of the links. This thorough topological characterization allows us to discriminate between our model and pared-down versions thereof, which capture some but not all of the above features of the yeast network. In this sense the model we present here is a minimal null model.

The focuses of both empirical and theoretical network-theory approaches to gene regulatory networks have been studies of the degree distribution [7], [8] and network motifs [27]–[30]. Barabasi and co-workers [31] have claimed that the global properties of gene regulatory networks of yeast (*Saccharomyces cerevisiae*) and *Escherichia coli*, as well as protein-protein interaction and metabolic networks, can be modeled by the preferential attachment [11], [20] rule and that these networks are scale-free, with the degree distribution having a scaling exponent [27] *γ*∼2.5. Smaller values for this exponent can also be found in the literature [8], [32]. Bergmann et al. [32] have suggested that the degree distribution might have a universal scale-free behavior independent of any particular organism. The claims of scale-invariance are typically based on linear fits to the log-log plots of the degree distribution data over narrow intervals of about two decades or less, and with imperfect agreement between the data and the fit. The careful analysis of Guelzim et al. [7] has revealed that the in- and out-degree distributions are rather different, with the former having an exponential-like decay and being confined to a much narrower range. Regarding the local organization of such networks, statistics of the various *n*-node motifs have been reported to be significantly different from randomized versions of the same network [27]–[30], and therefore assumed to be of functional or evolutionary significance.

It should be mentioned that the idea of using the matching of linear codes, as embodied in our sequence-matching rule, to model the satisfaction of a broad set of requirements for the binding of proteins to other molecules, is not entirely new. Complementarity of binary sequences (“bit-strings”) of fixed uniform length representing anticores and the antigens which “recognize” them have been employed in modeling immune networks in the early 1990’s [33], although the emphasis at this stage was more on the dynamics of small networks constructed in this way, than on their topological features. There have also been several earlier studies of models of gene regulatory networks on rather elaborate “Artificial Genomes” [34] based on various alphabets and matching rules [35]–[38], some of them coupled with the duplication and divergence model introduced by Wagner [39]–[41]. The results are not uniform and depend on the detailed assumptions made in the models.

The organization of the paper is as follows. In the Analyses section, we elaborate in some detail the idea of an information-theoretic approach to interaction networks. We explain the sequence-matching rule and discuss the biological input that goes into our model. In the Results section, our model simulations are compared with a thorough characterization of the gene regulatory network of yeast using the most comprehensive available data (see Table 1). Here we also present the results of comparisons with several, more restricted models, and with randomized networks. Our conclusions are presented under Discussion. The Methods section is reserved for more technical details.

## Analyses

### Information theoretic approach to interaction networks

The information-theoretic approach we would like to present in this paper is quite generic and promises to be widely applicable to systems which can be described in terms of networks of interacting nodes. In this approach, an interaction, represented as an edge connecting a pair of nodes, is established if and only if a number of more or less stringent constraints are fulfilled. The number and strictness of the constraints may be quantified as a certain amount of information, or code, that has to be shared between the two nodes. The topology of the interaction network is then determined by the distribution function of the required amount of shared information between the interacting nodes.

The way in which we model the shared information, corresponding to a set of constraints, is via a string-matching condition we have introduced earlier [16], [17]. In this “content-based model,” the condition for establishing a connection is that a code, represented by a string associated with one node, match, letter for letter, a sub-string of the code associated with another node. In this model, matching each successive letter will correspond to satisfying an additional constraint. The number of constraints can thus be mapped to the length of a string to be matched. The chance satisfaction of the constraints is smaller, the longer the strings, or the larger the alphabet from which they are constructed.

In fact, it can be shown that to a first approximation the probability that a random string of length *l*_1_ is included inside another random string of length *l*_2_ (*l*_1_≤*l*_2_) is given by [17],
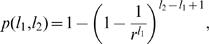
(1)where *r* is the number of letters in the alphabet from which the strings are chosen independently and with equal probability for each letter (1/4 for four nucleic acids, 1/2 for a binary code etc.). This approximation is valid for lengths *l*_2_−*l*_1_ smaller than or comparable to 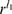, which amounts to the condition that correlations arising from multiple occurrences of the shorter string inside the longer one can be ignored. In fact, when 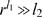 the above expression reduces to
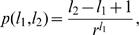
(2)which is the naive result obtained by treating the probabilities of a match of the shorter string along the *l*_2_−*l*_1_+1 positions on the longer string as independent. Also note that the average number of occurrences, 〈*n*〉, of the shorter randomly drawn string inside the longer one is given exactly [42] by the right hand side of Eq. (2). We see that Markov′s Inequality for integral valued random variables implies that Prob(*n*≥1)≤〈*n*〉 and re-expresses the fact that for 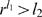 the probability of having more than one occurrence can be neglected.

To use a “lock and key” analogy to illustrate the idea of simultaneously satisfying a number of constraints, the first string may be regarded as the “key” combination that opens the “lock,” which in this case may be opened by more than one key (at most *l*_2_−*l*_1_+1 keys). The probability of a chance hit on one of the right combinations decreases exponentially with *l*_1_, as exp(−*l*_1_ ln *r*). Note that −*l*_1_ ln *r* is in fact the so called “Shannon information” [43] of a random “key,” selected from an alphabet of *r* letters.

We may easily compute the information [43] coded in a string of a given length, whose loci may have different sets of probabilities for encountering different letters. This quantity can then be used to define an effective length for a corresponding string of random letters. This is described in the Methods section.

This approach seems to be particularly well-suited to the description of gene regulatory networks, which operate on a cognate/cognate response element basis. TFs are the cognates, which bind the cognate response elements, i.e., the binding sequences (regulatory sequences) within the PRs of different genes. The “key” to the promoter region, so to speak, is the binding motif.

### Sequence matching model for the transcription regulatory network

**Figure 1:**
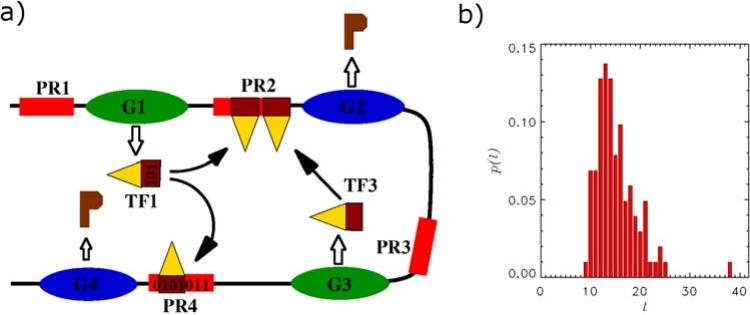
The model. (a) The mechanism of interaction between the genes as envisaged in our model. The genes are indicated by ellipses (green if they code transcription factors (TFs), blue otherwise), the TFs by triangles with the associated binding motif (regulatory sequence) in the box underneath. Non-TF proteins are symbolized by the “P” shape, and the promoter regions (PRs) upstream of each gene are shown as red boxes. Binding occurs if the binding motif exactly matches a subsequence in the PR, as is the case here at PR4. PRs in the model are typically much longer than depicted here. (b) Distribution of the amount of bit-wise information coded by each regulatory sequence recognized and bound by the 102 TFs in the yeast genome, compiled from the recently published data by Harbison et al. [9]. This distribution is adopted as the length distribution of the random regulatory sequences (“binding motifs”) in our model.

The nodes of our model network correspond to genes, only a small percentage of which code for transcription factors. With each node we associate a sequence, which represents the PR through which the corresponding gene may be regulated. With those nodes/genes coding TFs, we also associate a second sequence, uncorrelated with the first, representing the binding motif recognized by the TF. For simplicity we assume that there is only one transcription factor that is coded by each regulatory gene. (see Fig. 1a)

In our model the binding motifs and the PRs are represented as random binary sequences (thus, with *r* = 2), whose lengths obey different probability distributions. The TF binding motifs are typically short sequences with a narrow length distribution [3], [9], since a TF selectively binds 5–10 bases and not much more. A single TF can bind a number of similar sequences, and we have used the information content of the binding motifs representing these sequences in order to obtain a distribution of effective lengths (see Fig. 1b) for the randomly generated binary sequences representing our TF binding motifs. This is described in the Methods section.

For the PRs we make the assumption that the lengths are distributed in the same way as the lengths of the intergenic regions, obeying long tailed power-law distributions [18] whose exponent is the only free parameter in the model, and will be determined from a comparison of the topological features of the model and the experimental regulatory networks, as described in the next section. We have tested whether a simpler assumption regarding the distribution of the PR lengths could also suffice. Taking a constant PR length for all genes leads to similar qualitative behavior, although it performs worse when compared with actual yeast data (see supporting Text S2).

The amount of information coded in these randomly generated binding motifs and promoter regions thus constitutes the essential biological ingredient of our model and dictates the overall topology of the resultant networks.

The mechanism for establishing connections between nodes of the gene regulatory network is given by the string matching condition [16], [17] described above, between the binding motifs of the TFs and all possible uninterrupted subsequences of the PRs. The (directed) network of regulatory gene interactions is obtained by drawing a directed link from each TF-producing node A to all those nodes B, B′, B″,… whose PRs contain the binding motif associated with the TF coded by node A (see Fig. 1a and supporting Text S3).

## Results

### The yeast transcriptional regulatory network

To make a quantitative comparison with the yeast transcriptional regulatory network possible, we choose the total number of genes and the proportion of those genes coding for transcription factors (4.8%, see Table 1) in conformity with the Yeastract data set [5].

The length distribution of the binding motifs in the model genome was derived from the yeast data provided by Harbison et al. [9], where the motifs were reported as letter sequences comprising the symbols for the four bases {ATGC}, or the symbols {YMKRSW} signifying a preference for any two out of four bases, etc., with the corresponding lower case letters indicating a lower confidence level. In order to account for such variations in the information content of the motifs, we assigned two bits to each of the letters {ATGC} appearing in the motif, signifying a high information content at that position, one bit to each of the letters {atgcYMKRSWymkrsw} and zero bits otherwise. The length of the bit sequence obtained in this way roughly corresponds to the amount of shared information, measured by the Shannon entropy [43], required for the binding of the TF. Performing this calculation (see the Methods section) for each TF [9], we obtain the length distribution shown in Fig. 1b.

We assume that the lengths of the PRs follow a power law distribution similar to that of the intergenic regions [18], with 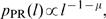
(3)where 0≤*μ*≤2. We also stipulate that *l* is restricted to the interval *l*_min_≤*l*≤*l*_max_, where *l*_min_ coincides with the peak of the motif-length distribution shown in Fig. 1b, while *l*_max_−*l*_min_+1 = 250. In this choice we are guided by the finding [9] that most of the probability for encountering a TF binding site is contained within a window of 250 base pairs (bps) located approximately 100 bps upstream of a gene. Note that the 250 bps window does not double as we move from the four-letter alphabet to a binary one, because the matching probabilities and the total number of positions at which the TFs may bind are required to remain invariant under this transformation. This amounts to preserving the average number of matches of a four-letter RS of length *l*_1_ in a four-letter PR of length *l*_2_, which is given by the right hand side of Eq. (2) (see the Analyses section). The denominator in Eq. (2), namely 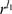, remains invariant under the transformation from a four- to a two-letter alphabet, as can be seen from the Methods section. For the numerator of Eq. (2) to also remain unaltered, *l*_2_ should be changed such that *l*_2_−*l*_1_ remains approximately unchanged; in particular, for *l*_1_≪*l*_2_, it means that *l*_2_ should be left unchanged.

Once the shape of the length distribution of the binding sequences and the functional form, as well as the support, of the length distribution of the PRs have been fixed through the available biological data, the only remaining adjustable parameter in our model is the exponent *μ* of the power law distribution of PR lengths, *p*_PR_(*l*).

Clearly, the length distribution for the PRs must be tested against null assumptions, and this we do in Text S2. We find that, once the form of the distribution has been chosen as in Eq. (3), any value of *μ* within the interval [0,2] performs reasonably well, while, say, fixing all the PR lengths to be identical gives markedly different results.

**Figure 2:**
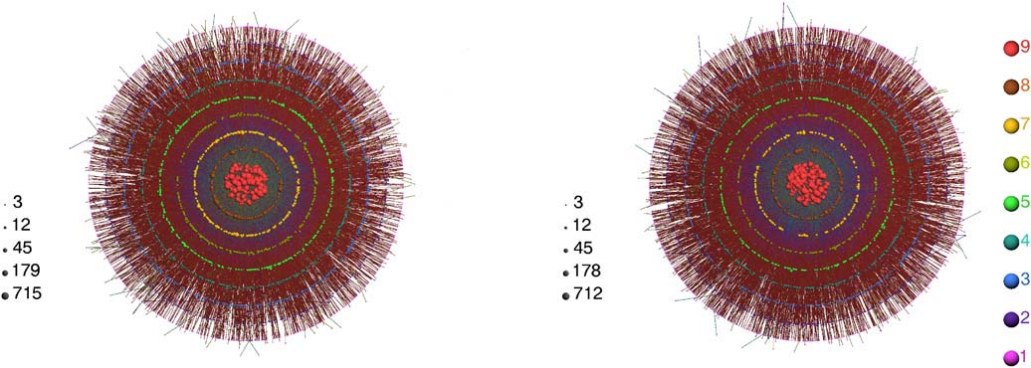
The hierarchical structure of the TRN as revealed by the k-core decomposition. The *k*-core visualization of a single realization of our model network (Left) obtained with the visualization tool lanet-vi (http://arxiv.org/abs/cs.NI/0504107) The length distribution exponent of the PR sequences has been adjusted to *μ* = 0.1 to match the number of *k*-cores to that obtained from Yeastract data (Right). Dots represent the nodes of the network, while edges between nodes depict connections. Nodes belonging to different *k*-shells are indicated by different colors (on the right hand side) and are arranged around concentric circles, whose average radius decreases with *k*. In particular, a node of a given shell is placed just inside (outside) the corresponding circle, if it is preferentially connected to lower (higher) *k*-shells. The sizes of the dots indicate the degree of the respective nodes; see legends to the left of the figures for representative sizes.

**Figure 3:**
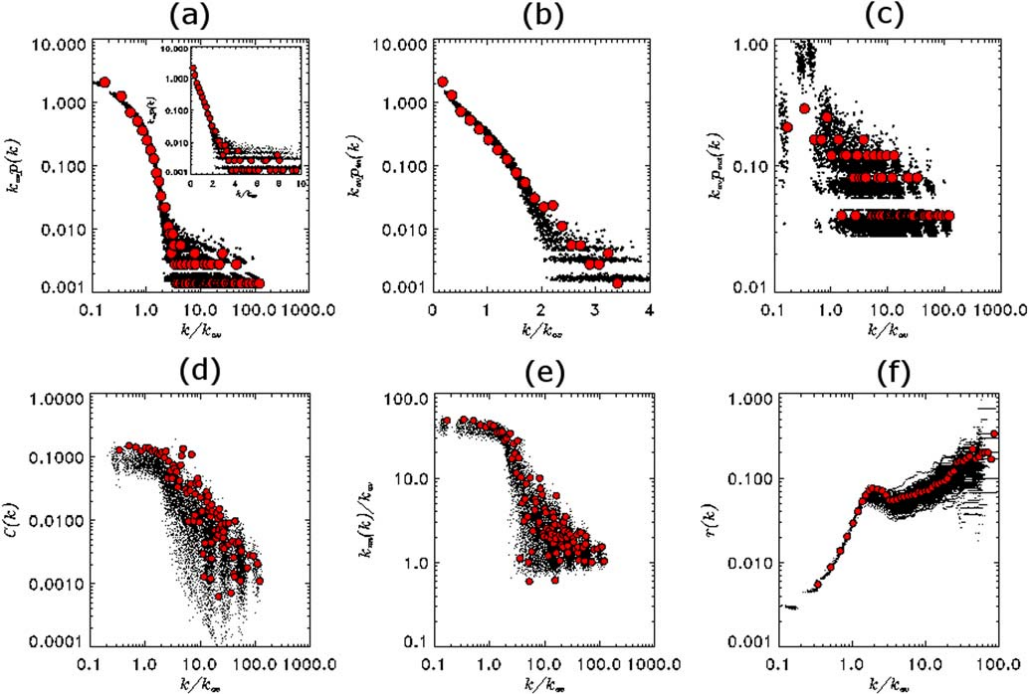
Topological features. Above: Degree distributions extracted from the Yeastract [5] data (red circles), superposed on the corresponding degree distributions of one hundred realizations of the model network (black dots). From left to right, (a) The total degree distribution with an inset showing a log-linear plot for *k*/*k*_av_≤10, where one may observe that both the model and the data points almost fall on a straight line. (b) The in-degree distribution plotted on a semi-logarithmic scale. (c) The out-degree distribution plotted on a log-log scale. The axes are scaled by the appropriate average total degree in order to factor out sample-to-sample fluctuations in the network size. Below: Comparison of (d) the clustering coefficient *c*(*k*), (e) the typical degree-degree correlations between neighboring nodes *k*_nm_(*k*), and (f) the rich-club coefficient *r*(*k*), from left to right, for 100 realizations of the model (black dots) and the Yeastract data (red circles).

In order to optimize the value of *μ*, we could compare all the available topological characterizations of randomly generated model networks obeying the constraints on the number of nodes, the length distribution of the binding sequences, for different values of 0≤*μ*≤2, with those of the yeast TRN. It is obviously desirable, however, to find one number to compare with experiment, rather than, say, the whole degree distribution or the degree-degree correlation function *k*_nm_(*k*). In fact, once *p*_PR_(*l*) is chosen to be of the power-law form given in Eq. (3), *then* choosing *μ* so that *k*_max_, the maximum number of *k*-cores [19] of the model, coincides with that of the network obtained from one of the yeast data sources, is sufficient for the rest of the topological features of the respective networks to fall right on top of each other, as shown in Fig. 2 and Fig. 3.

We build our ensemble of model networks starting from 6000 nodes. Out of these, almost all the nodes with nonzero degree belong to the largest connected component (see Table 1), whose size depends on the value of *μ*. The analyses for the degree distributions, the rich-club coefficient and the *k*-core structure have been performed for all the nodes with nonzero degree, while the clustering coefficient and the degree-degree correlation have been calculated on the largest connected component.

In Fig. 2, we show the *k*-core visualization (obtained by means of the LaNet-Vi tool, http://arxiv.org/abs/cs.NI/0504107) of one realization of the model network (left) and the Yeastract [5] data (right). Here *μ* has been fixed to 0.1, making the mean and the mode of *k*_max_ for the model ensemble to coincide with the value we compute from the Yeastract [5] database, at *k*_max_ = 9 (see Text S2 for details). Both the model and the experimental network exhibit a highly hierarchical structure with a nested sequence of *k*-shells and an almost exclusively radial arrangement of the edges. The distinct hierarchical organization of the edges is not very sensitive to the precise value of *μ*, while the total number of shells decreases as *μ* increases. (see Text S2)

In Fig. 3, we report our results for the in-, out- and total degree distribution [19]–[22], the clustering coefficient [19]–[22], the degree-degree correlation [23], [24] and the rich-club coefficient [25], [26] (the precise definitions of which are given in the Methods section), with the choice of *μ* = 0.1, i.e., the topological features displayed in Fig. 3 are obtained without any further adjustment of *μ*. Results for the yeast TRN, which we have extracted from the Yeastract [5] data have been superposed on the scatter plots of one hundred independent realizations of randomly generated model networks with identical parameters.

The total degree distribution is obtained by ignoring the directionality of the interactions and is generally different from the superposition of in- and out-degree distributions. In Fig. 3a, Yeastract [5] data for the degree distribution is shown on top of a scatter plot obtained by superposing the results of the ensemble of model networks. The average total degree of the yeast TRN extracted from Yeastract [5] is *k*_Yeastract_ = 5.9, while that found from our model is *k*_Model_ = 6.9 with the standard deviation *σ_k_* = 0.7. In Fig. 3b, we exhibit the in-degree distribution obtained from the Yeastract [5] data, and the corresponding scatter plot.

The out-degree distribution of the yeast and model networks has a rather large scatter of points due to the relatively small number of TFs. Comparing with the scatter plot obtained from one hundred realizations, we find again that the actual yeast data falls within the boundaries set by the model ensemble (Fig. 3c).

In Fig. 3(d,e,f), we report the three topological coefficients, namely, the clustering coefficient, the degree-degree correlation and the “rich-club” coefficient, that go beyond degree-distributions in characterizing the network. The agreement is extremely good; in particular, the shoulder observed in the “rich-club” coefficient in Fig. 3f, a feature common to both gene regulation and protein-protein interaction networks [26], is captured accurately in our model. The average clustering coefficient for yeast is *C*_Yeastract_ = 0.08, while *C*_Model_ = 0.06 with the standard deviation *σ_c_* = 0.01.

We have compared the topological properties of our model networks with those of the TRN of yeast obtained from the different sources listed in Table 1. The number of interacting nodes in these data sets vary between 2884 and 4252, while the number of interacting pairs vary between 6441 and 12530. All the data sets give rise to statistically similar topological features as can be seen by superposing the data points from all different sources on the scatter plots obtained from our model (see Fig. 1 of Text S4). The topological features computed from all the different data sets are reproduced faithfully with a single *μ* optimized with respect to Yeastract only.

### Comparison with randomized results and null-null models

In this section we briefly discuss comparing the topological features of our model with randomized versions thereof, and with pared-down models which incorporate only a few elements of our model, selected to mimic only certain phenomenological properties of the target network, but not incorporating either the full biological information in the form of realistic length distributions, or the full sequence matching rule. These pared-down versions of our model are designed to act as null-hypotheses with respect to the null-model we have constructed, and therefore we will call them “null-null” models. The details of the computations and figures are presented in Text S5.

To check the significance of our results, we have subjected both the real and the model networks to a rewiring of the edges while *i)* keeping the degree distributions fixed while ignoring the directionality and *ii)* conserving the directionality so that the in- and out- degree distributions are kept fixed separately.

Randomly exchanging the endpoints of pairs of edges while keeping only the total degree of each node fixed, destroys the hierarchical structure, and the topological features computed here are radically altered. Rewiring the model network and yeast TRN while keeping the in- and out-degree of each node separately invariant by conserving the directionality of the edges, leaves all of the topological features displayed in Figs. 2 and 3 practically unchanged.

It should be noted that, in our model the lengths (and the contents) of the binding sequence with which we label the TF and the PR associated with the same node are assigned independently of each other. Thus there is no correlation between the in- and out-degrees of a given node. Our model is, therefore, a null model in this respect. Invariance of the topological features under a random rewiring which conserves the in- and out- degree distributions, suggests that the in- and out-degree distributions together are able to determine *all* of the global topological features of the network in question. The achievement of our model is that it does not have to import these degree distributions from empirical data; it is able to capture them by means of the string-matching rule and the length distributions of the TF and PR sequences appropriate to the organism under study.

To double check our randomization procedure, we have simulated an ensemble of configuration model networks [44], whose in- and out-degree sequences are extracted from different realizations of our content-based model. We connect the nodes randomly, while respecting the in- and out-degree assignments (see Text S5 for details). The topological features of the resulting networks are indistinguishable from the model ensemble.

To see whether we could reproduce certain features of the yeast TRN using only the fact that there are two types of nodes in this network, those coding for TFs, and others that do not, we constructed an Erdös-Rényi type of null-null model, where out-edges connect the TF-coding nodes to randomly picked nodes in the network with a probability *p* given by the density of edges on the yeast TRN. The resulting network is a superposition of two Erdös-Rényi random graphs. The results are quantitatively different from the yeast TRN in all respects, as shown in Text S5; however, qualitatively, they are somewhat reminiscent of the yeast network.

A coarse-grained, or mean-field, version of our model is obtained if, instead of the fluctuations coming from the chance coincidence of individual strings, one takes the ensemble averaged probability for a matching to occur between strings of given lengths, as in Eq. (1). This can be thought of as a hidden-variable model [45], where, instead of just the two types of nodes considered above, one has a superposition of a whole spectrum of Erdös-Rényi networks, with the connection probabilities *p*(*l_i_,k_j_*), between pairs of nodes *i* and *j*, with binding motifs and PRs of length *l_i_* and *k_j_*. Simulation results of this null-model are presented in Text S5.

The fidelity of the mean-field version to the yeast TRN is indistinguishable from that of the full content-based model. This gives us confidence that analytical calculations of ensemble averaged properties are quite meaningful. It should be remembered that *i)* the length distributions of the PR or binding strings have been extracted from empirical data using our information-theoretical approach to the binding specificities of the binding motifs, and *ii)* that the connection probabilities *p*(*l,k*) were derived from the string-matching condition.

## Discussion

Our results support our hypothesis that the topology of the TRN is predominantly determined by the interactions between the TFs and the response elements. In our model these interactions are schematized via the sequence-matching rule for the sharing of information between the cognates/cognate response elements. The close structural similarity between the model and the real yeast transcriptional regulatory network, with respect to a diverse set of criteria shows that they are part of the same statistical ensemble of networks [46]. This observation is further supported by a comparison of the frequency of various triangular network motifs (not to be confused with the “binding motifs” in the text), as provided in Text S1.

It should be noted that the present approach could also be adapted to model the topology of the gene regulatory network of *E. coli*, by taking into account the fact that in the prokaryotic genome the genes are organized into operons, each operon being regulated by a single promoter region. Work on this problem is in progress.

The sequence-matching rule should be viewed as an information-theoretical constraint, where the interaction between two genes requires the fulfillment of a set of conditions which we symbolically represent as the matching of two random sequences. The more stringent the prerequisites of the interaction, the longer is the random binding sequence that is to be matched. The length of the PR establishes the size of the phase space in which the motif is to be sought. The properties of the network are then determined by the distributions obeyed by the lengths of the binding motifs as well as the promoter regions.

Interpreted within an information-theoretical framework, our model has sufficient generality to accommodate other interactions based on constraint satisfaction mechanisms, such as protein networks, where the interactions are dictated by certain steric and chemical conditions.

The topological features of the networks investigated here, and shown to be shared by the yeast transcriptional regulatory network, strongly point to the possibility that these networks did not have to be assembled from scratch, but rather emerged spontaneously, given any sufficiently long, complex linear code, and a mechanism for the transcription of some of its subsequences into molecules (proteins) that in their turn have an affinity for parts of this code and bind it. That the length distribution of these sequences (and not their contents) is sufficient to reproduce the topological features investigated here, indicates a certain level of robustness to point mutations.

Our model provides a means to roughly predict the parameters of the length distribution of the regulatory sequences in other organisms (assuming, e.g., a Gaussian form), given their regulatory network topology. Even more significantly, it may help identify the biologically relevant topological features that may have been acquired in the course of evolution, which require more specific information than the length distributions to be pinpointed, and hence are not captured by our model.

## Methods

### Bit-wise information content

In this model we have assumed that the information content of a sequence can be computed as the sum of the information content of each letter in the sequence, i.e., that the letters are not correlated, although their relative frequencies of occurrence may depend on their position. We have moreover, assumed that positions within the sequence have equal significance, i.e., the maximum amount of information which can be contained in any position within the sequence is uniform.

In a given sequence of length *L*, with letters chosen from an alphabet of length *r*, the information content, which is the negative of the Shannon entropy [43], is given by 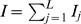 with
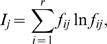
(4)where *f_ij_*, *i* = 1,…,*r* are the relative frequencies of the different letters at each position *j* in the sequence. Note that *I_j_* = 0 if we know for sure that a certain (e.g., *i*th) letter and no other, will appear (in which case the relative frequency *f_ij_* = 1 and *f_i_*_′*j*_ = 0, for *i*′≠*i*). Thus, Shannon information is the amount of information which we receive from a signal over and above what we already knew about the system. Let us define a relative Shannon information, *R* = Σ*_j_R_j_*≡Σ*_j_I_j_*+*L* ln *r*, which is the difference between *I* and the Shannon information communicated by a signal composed from an alphabet with equi-probable letters. This is the definition of information content which we will use. (Note that the so called log-odds matrix, where one takes ln(*f_ij_*.*p_ij_*), where *p_ij_* is the so called “background” probability, here taken to be uniformly equal to 1/*r*, is related but not quite the same, since the natural logarithms are not multiplied in this case by the respective frequencies in computing the information content. The Kullback-Leibler (information) divergence [47] is another closely related measure, which, for a uniformly random background distribution, is identical to the relative information defined here.)

For a four-letter alphabet, the length increment which the *j*th member of the sequence will contribute, is, 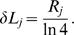
(5)


The bit-wise information content of a sequence is the number of binary digits 0,1, needed to code the same amount of information. Thus the bit-wise length increment of the same character will be 
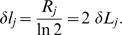
(6)


However, *δl_j_* is not, in general, an integer (neither is *δL_j_*). Therefore a coarse graining, which entails a certain amount of arbitrariness, is called for. In the context of transcriptional gene regulation, the binding sequences are reported in Harbison et al. [9] in an enriched alphabet, with upper case letters indicating a high preference, lowercase letters a weaker preference, with the letters {ATCG} for the nucleic acids, the “ambiguity codes” {SWRYKM} for pairs of letters out of the four, i.e., S = C or G, W = A or T, R = A or G, Y = C or T, K = G or T, M = A or C, the codes {HBVD} for different triplets out of the four letters, and finally, N indicating “no preference.” Plotting *δl_j_* on the real line for all the empirically encountered binding motif elements, we find that these values fall into distinct clusters over the interval [0,2], grouped according to their codes within this enriched alphabet. Thus, for example the interval (1.04,2] corresponds to {ATGC}, (0.3,1.04] to {actgswrykm}, and [0,0.3] to the letter {n}. We accordingly make the following choice for a coarse grained, integer valued Δ*l_j_*, 
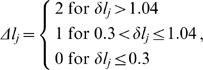
(7)with the bit-wise length of a sequence being finally given by Σ*_j_*Δ*l_j_*.

Note that, in computing the relative information content, instead of assuming equal probabilities for the different letters of the alphabet, we could have taken the approximate proportions of 0.3, 0.3, 0.2 and 0.2 of the letters {ATCG}. Then, the Shannon information for a random sequence composed of these letters with the aforesaid probabilities would have been −1.366 rather than −1.386 = −ln4. This introduces a uniform shift of the relative information content by a small increment, 0.02. The length increment per character thus gets uniformly shifted by 0.02/ln2 = 0.029. Since the shift is uniform, taking the same criteria for the coarse graining procedure yields an identical spectrum of bit-wise length increments as the one obtained with equi-probable random letters for the nucleic acids.

### Topological quantifiers of complex networks

The degree *k* of a node is the number of nodes connected to it. When the graph is directed, one distinguishes in-, out-, and total degrees of a node, with their corresponding distributions. In the measures below we have ignored the directionality of the network.

The clustering coefficient is given [19]–[22] by the formula: 
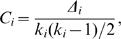
(8)where Δ*_i_* is the number of triangles that contain node *i*. The quantity *C*(*k*) plotted in Fig. 3d is the average of *C_i_* over the nodes with degree *k*.

The degree-degree correlation function *k*_nm_(*k*) is [24], [23]

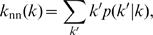
(9)where *p*(*k*′|*k*) is the conditional probability that a node with degree *k* is connected to a node with degree *k*′.

The “rich-club” coefficient [25], [26] *r*(*k*) is the total number e*_>K_* of edges connecting nodes with degree greater than *k*, normalized by the maximum possible number of such connections, 
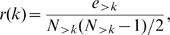
(10)where *N_>K_* is the total number of nodes with degree greater than *k*.

The hierarchical organization of a network is revealed by the *k*-core decomposition, which performs a successive pruning on the least connected vertices of a network [19]. At each step one removes all nodes with a degree less than *k* along with their edges and continues in this manner until all nodes have at least degree *k*. The remaining nodes constitute the *k*-core. Next, *k* is incremented by one, and the process is repeated until no nodes are left. The *k*-shell is defined as the set of nodes that belong to the *k*-core, but not the (*k*+1)-core. The *k*-core decomposition provides a highly detailed topological characterization of the network, if, besides the total number of shells, the distribution of the nodes over the shells and inter- and intra-shell connectivity [48] are also taken into account. (see Text S2)

## Supporting Information

Text S1 Comparison of 3-Motifs for the Model and the various Yeast Data Sources
(0.05 MB PDF)

Text S2 Qualitative and Quantitative Aspects of the k-core Structure: Choosing the Length Distribution of the Promoter Regions
(0.40 MB PDF)

Text S3 Ranking of Overlapping Sets of Regulated Genes and Sequence Inclusion
(0.02 MB PDF)

Text S4 Comparison of the Model with the Yeast Data from Different Databases
(0.04 MB PDF)

Text S5 Randomization Procedures and Null-Null-Models
(2.19 MB PDF)
