# Supplementary material for: The Information Coded in the Yeast Response Elements Accounts for Most of the Topological Properties of Its Transcriptional Regulation Network
Source: PLoS One. 2007 Jun 6;2(6):e501. doi: 10.1371/journal.pone.0000501 (PMC1876808; doi:10.1371/journal.pone.0000501)
Supplement: Text S1 — Comparison of 3-Motifs for the Model and the various Yeast Data Sources (0.05 MB PDF) [file pone.0000501.s001.pdf]

Text S1

Comparison of 3-Motifs for the Model and the various Yeast Data Sources

Supporting Information for

The Information Coded in the Yeast Response Elements Accounts for most of the Topological Properties of its Transcriptional Regulation Network

Duygu Balcan, Alkan Kabakçıoğlu, Muhittin Mungan, Ayşe Erzan

Using the motif finder tool `mfinder` *, we have compared the number of 3-motifs † in one realization of our model network with those found in the yeast transcriptional regulatory network obtained from the data sources of Table 1 in the main text. We have also compared the number of 3-motifs in each of these networks with the corresponding averages obtained from 100 random rewirings such that in- and out-degree of each node remained unchanged. There are 13 different 3-motifs shown in Fig. 1, along with their motif identification numbers, given as "id$n$."

Since different networks obtained from the different databases, are of different sizes, the total number of 3-motifs in each varies considerably, as shown in Table 1 in this Supplement. Therefore, in each case we have calculated the fraction of the number of occurrence of a given motif as parts in 1000, in comparison to the total number of 3-motifs in that particular network. Table 2 shows the relative frequency of occurrence of the different motifs in the network and its corresponding randomized forms. Motifs with a relative frequency less than one part in 10000 have been excluded. We find that the relative frequency of the different motifs are more or less uniform over the networks obtained from the four data sources for yeast, and the relative frequency of the motifs obtained from our network model are overall comparable with those obtained from the four different sources for yeast data. Compared with all four data sources, motif 6 is somewhat over-represented in our model, while motifs id12, id38 and id46 are under-represented. Given the extremely large - close to two million - number of 3-motifs, we have assumed their frequencies to be self-averaging.

Comparing these results with the randomized networks, we see that for the ensemble of model networks, the relative frequencies of the different 3-motifs for the randomized networks are overall comparable to those from the non-randomized ones; the randomized networks yield average motif frequencies that are within one standard deviation (shown in parentheses) of the non-randomized ones. On the other hand the results obtained by randomizing the yeast data from the different sources seem to suggest that the average motif frequencies for the randomized and non-randomized

networks differ by much more than one standard deviation, in particular for motifs 14, 36, 38, and 46. This finding points to rather subtle differences, residing in local features, between the regulatory network topology and that obtained via sequence-matching from a random model genome.

It should be noted that in our null-model, the two sequences representing the TF coded by a gene and the PR are completely independent. Thus, to introduce local correlations one needs to go at least to the 4-motifs, where, for example the existence of the directed bonds $1 \rightarrow 2$, $1 \rightarrow 4$ and $3 \rightarrow 4$ induce a nontrivial conditional probability for the existence of the bond $3 \rightarrow 2$.

[*] Freely available from `www.weizmann.ac.il/mcb/UriAlon/`

[†] Shen-Orr SS, Milo R, Mangan S, Alon U (2002) Network motifs in the transcriptional regulation network of *Escherichia coli*. Nature Genetics 31: 64-68; Milo R, Shen-Orr S, Itzkovitz S, Kashtan N, Chklovskii D, Alon U (2002) Network motifs: simple building blocks of complex networks. Science 298: 824-827.

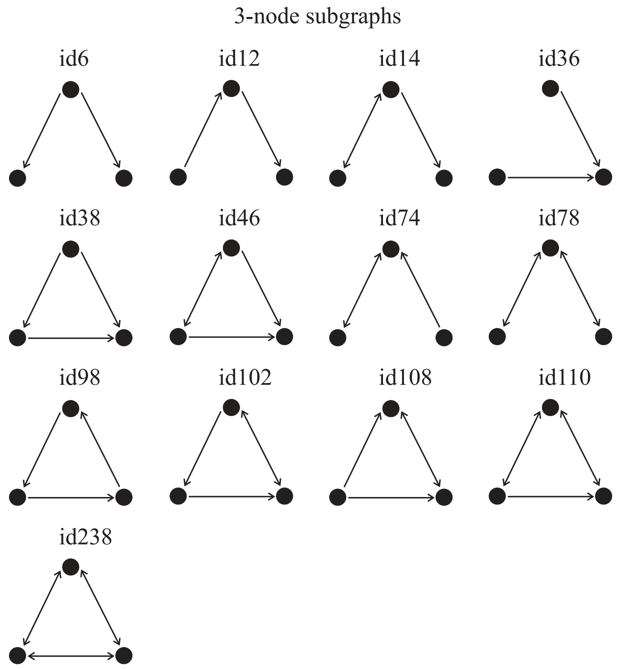

Figure 1: The set of 13 possible 3-motifs along with their ids.

Table 1: Number of 3-motifs found in the yeast transcriptional regulatory network obtained from the data sources of Table 1 (main text)

Source	3-motifs
Model	1858311
Yeastract[a]	1607319
Luscombe[b]	531170
Fraenkel[c]	438504
Kırdar[d]	746728

[a] Teixeira MC, Monteiro P, Jain P, Tenreiro S, Fernandes AR, et al. (2006) The YEASTRACT database: a tool for the analysis of transcription regulatory associations in *Saccharomyces cerevisiae*. Nucl Acids Res 34: D446-451.

`http://www.yeastract.com`

[b] Luscombe NM, Babu MM, Yu H, Snyder M, Teichmann SA, et al. (2004) Genomic analyis of regulatory network dynamics reveals large topological changes. Nature 431: 308-312.

`http://sandy.topnet.gersteinlab.org/index2.html`

[c] Lee TI, Rinaldi NJ, Robert F, Odom DT, Bar-Joseph Z, et al. (2002) Transcriptional regulatory networks in *Saccharomyces cerevisiae*. Science 298: 799-804.

`http://fraenkel.mit.edu/Harbison/release_v24/bound_by_factor/`

[d] Kınıkoğlu B, et al. (2006) Identifying perturbation responsive transcription factors in *Saccharomyces cerevisiae*. submitted to Yeast.

private communication

Table 2: The relative frequencies of 3-motifs with ids 6-74, found in the model network as well as the four data sources listed in Table 1, specified as parts in 1000. The second line for each data source shows the resulting average frequencies obtained from 100 random rewirings that keep the in- and out-degree of each node fixed. The corresponding standard deviations are in parentheses.

Source/id	6	12	14	36	38	46	74
Model	970.26	11.67	1.28	15.65	1.05	0.07	0.02
Ran.	970.28(0.06)	11.61(0.06)	1.31(0.02)	15.61(0.06)	1.11(0.06)	0.05(0.01)	0.02(0.00)
Yeastract	964.94	17.71	1.34	13.97	1.85	0.14	0.04
Ran.	964.48(0.09)	17.96(0.09)	1.56(0.02)	14.33(0.09)	1.58(0.09)	0.03(0.01)	0.04(0.00)
Luscombe	958.15	24.88	0.45	14.76	1.62	0.12	0.02
Ran.	957.63(0.10)	25.17(0.11)	0.68(0.01)	15.17(0.10)	1.31(0.10)	0.01(0.00)	0.02(0.00)
Fraenkel	951.43	24.41	2.84	17.59	2.91	0.72	0.08
Ran.	949.10(0.12)	25.62(0.12)	4.02(0.04)	19.44(0.13)	1.57(0.12)	0.13(0.02)	0.10(0.01)
Kırdar	955.60	20.44	3.25	18.46	1.87	0.31	0.06
Ran.	954.68(0.09)	20.97(0.09)	3.66(0.03)	19.21(0.09)	1.30(0.09)	0.10(0.02)	0.07(0.00)
