# Supplementary material for: The Information Coded in the Yeast Response Elements Accounts for Most of the Topological Properties of Its Transcriptional Regulation Network
Source: PLoS One. 2007 Jun 6;2(6):e501. doi: 10.1371/journal.pone.0000501 (PMC1876808; doi:10.1371/journal.pone.0000501)
Supplement: Text S2 — Qualitative and Quantitative Aspects of the k-core Structure: Choosing the Length Distribution of the Promoter Regions (0.40 MB PDF) [file pone.0000501.s002.pdf]

Text S2

Qualitative and Quantitative Aspects of the k-core Structure: Choosing the Length Distribution of the Promoter Regions

Supporting Information for

The Information Coded in the Yeast Response Elements Accounts for most of the Topological Properties of its Transcriptional Regulation Network

Duygu Balcan, Alkan Kabakçıoğlu, Muhittin Mungan, Ayşe Erzan

In this supplement we would like to discuss certain qualitative and quantitative aspects of the k-core decomposition, how these are related to the other global topological features of the network and how we have used them in deciding upon a length distribution for the PR regions. We would also like to warn, here against an undue emphasis on the visual appearance of the k-core decomposition, which may obscure more subtle differences between networks with respect to such properties as the distribution of the nodes over the different k-shells.

Invariance of the topological features under a random rewiring which conserves the in- and out-degree distributions, suggests that the in- and out-degree distributions together are able to determine *all* of the global topological features of the network in question. (see main text, the Results section) Capturing the k-core visualization correctly is a necessary but not sufficient condition in modelling a real network. The k-core visualization may aid in a quick elimination of certain candidate models, and may amplify certain subtle differences, but in the case of our content-based model, the choice of the appropriate length distribution for the target strings cannot be made on the basis of the k-core visualization alone.

This supplement is organized as follows. First we present, for sharp contrast with Fig. 2 in the main text, two k-core plots, one pertaining to a simple sequence matching model with only one sequence associated with each node*, and the other corresponding to the preferential attachment model of Barabasi and Albert**, which has been proposed as a model for gene regulation networks. Then we present the detailed considerations leading to our choice of the exponent μ in the power-law distribution for the PR lengths, the quantitative analysis of the k-core decomposition of the resulting network, and comparison to that of the TRN of yeast. For comparison, we discuss the effect of varying μ over its whole range, and we show the results for the global topological features of the model networks, again superposed with those of Yeastract, for $\mu = 2$. Finally, we pose the

question whether a different length distribution altogether, such as fixing the PR lengths at some relatively large value, could not have yielded similar agreement with yeast data. As a worst-case example we present a k-core visualization which agrees very closely with that of yeast, but which sharply diverges in its quantitative k-core structure and other topological features.

k-core plots of the simple sequence-matching model and the Barabasi-Albert model. In Fig. 1 we show, the k-core structure of two complex networks, the Balcan-Erzan [*] model, employing a string-matching connectivity condition with only a single string associated with each node, and the preferential attachment model of Barabasi-Albert (BA) [**]. In the Balcan-Erzan network we find 54.5 % of the nodes in the highest k-shell ($k = 193$), and an extremely fast decay of the population down to zero at shell number 77. In the BA network, there are only three shells, with 99.9 % of the nodes in the second shell, and the third shell being just the four completely connected set of nodes from which the network is grown. Note the absence of a well-defined hierarchical structure.

Determining the value of μ. We find that employing a power law form, $p(l) \sim l^{-1-\mu}$ for the length distribution [††] of the promoter regions (PRs) in the present model, leads to the hierarchical organization that can be seen in Fig. 2 of the main text, with the connectivities essentially being between the innermost core and the outer k-shells. This feature is not sensitive to the precise value of μ.

To choose the value of μ to be used in our simulations, we proceed as follows: For different values of μ, we generate an ensemble of one hundred realizations of our model network, with $N_G = 6000$ genes in total, as in the yeast genome. The value of $\mu = 0.1$ yields the greatest proportion of model networks (36 out of 100) with $k_{\max} = 9$, coinciding with that for yeast. Moreover, for $\mu = 0.1$, the distribution of $k_{\max}$ for different realization of the model network is symmetric about the mode.

Without any further adjustment of the value of μ, we find that the size of the connected network relative to the size of the whole genome (in number of nodes) is in the right ballpark. On the average, 4167 genes out of the total contribute to the $\mu = 0.1$ model regulatory network, to be compared with 4252 (see Table 1 in the main text) for yeast. Out of these 4167 genes, we choose 4.8 %, namely 202 genes, to be TF-coding genes. They end up taking part in a total of 14365 interactions, again on the average. The corresponding values for the yeast regulatory networks reported in the available databases are given in Table 1 (main text).

Quantitative analysis of the k-core decomposition provides a highly detailed topological characterization of the network, with the total number of shells, the distribution of the nodes over the shells and inter- and intra-shell connectivity.[†] There is detailed quantitative agreement between the k-core decomposition of the Yeastract network and our model, for $\mu = 0.1$, as can be seen from Fig. 2, where we show the population of each shell and the distribution of the links among different shells. Both for the yeast data and the model, the great majority of the links connect the innermost shell with the others.

The results reported in Fig. 2 may be compared with those of Carmi et al.[†] for the internet. The shell population dependence on k, namely $\sim k^{-1}$ is much weaker than that found for the internet Autonomous Systems. The general trend of the proportion of links out to the crust, into the core and in the same shell, are similar. However the identical exponential growth in the number of links connecting any given shell to shells deeper and deeper in the core-structure, is a strongly distinguishing feature of yeast and the model network which we are considering here.

Comparison with other values of μ. We have varied μ within the range $-0.5 \leq \mu \leq 2.5$, to see how far this affects the topological features of the model network qualitatively and quantitatively (see Table 1 for the ensemble averages of the number of nodes and edges of model network realizations for different values of μ). For $\mu > 2$ the distribution is not fat tailed (Almirantis and Provata [††] suggest $0 \leq \mu \leq 2$) and for smaller values the fluctuations would totaly dominate. (Note that the distribution stays normalizable since the range of l is finite.) We find that the relatively larger μ values result in fewer k-shells, with the average value of $k_{\max}$ ranging from 10.61 to 3.27 for $-0.5 \leq \mu \leq 2.5$ (see Fig. 3). The plots corresponding to Fig. 2c are successively truncated from the left as μ is increased, so that the hierarchical structure of the k-cores is, nevertheless, preserved throughout this range of μ values.

In Fig. 4 we show the topological features of the model network, computed for the relatively large value of $\mu = 2$. The figures here are qualitatively somewhat similar to Fig. 3 of the main text, although the quantitative agreement achieved for $\mu = 0.1$ is lost. For the lower value of $\mu = 0$, the behavior is very similar to that for $\mu = 0.1$, so we have not displayed it here. It should be remarked that the degree distribution is much less sensitive to the change in μ as compared to the clustering coefficient, the degree-degree correlations and the rich-club coefficient.

A null-hypothesis for the length distribution of the target sequences. Since there is a degree

of arbitrariness in the way we have assumed that the lengths of the promoter regions (PRs) should follow a power-law length distribution as do the intergenic regions[††], we decided to test the null-hypothesis that they are all of the same length, L.

A careful screening of different L values reveals that e.g., for $L = 50$, the degree distributions are qualitatively similar to those shown in Fig. 3 of the main text, with a slightly faster decay of the in-degree distribution. The k-core decomposition has the same hierarchical appearance as in Fig. 2 of the main text, but with $\langle k_{\rm max} \rangle = 4.35$. Nevertheless, the distribution of the nodes over the different k-shells, follows approximately the same k^{-1} decay as found before.

For larger L, we find that a peak forms in the in-degree distribution and moves to the right as $L > 100$, while for $L = 200$ one may indeed observe two humps. The number of k-shells at this point is $\langle k_{\rm max} \rangle = 13.5$ The hierarchical organization of the connectivity is preserved, although the distribution of the nodes over the different k-shells becomes non-monotone for $L \geq 100$.

Extrapolating between these two extremes in the number of k-cores, it is easy to find that for $L = 127$, the k-core plot shown in Fig. 5 almost coincides exactly with Fig. 2 of the main text, with $k_{\rm max} = 9$. However, the detailed qualitative and quantitative agreement with the topological features of the yeast network is lost, as shown in Fig. 6 and Fig. 7.

[*]Balcan D, Erzan A (2004) Random model for RNA interference yields scale free network. Eur Phys J B 38: 253-260.

[**] Albert R, Barabasi A-L (2002) Statistical mechanics of complex networks. Rev Mod Phys 74: 47-97.

[†] Carmi S, Havlin S, Kirkpatrick S, Shavitt Y, Shir E (2006) MEDUSA - New Model of Internet Topology Using k-shell Decomposition. cond-mat/0601240. arXiv preprint.

[††] Almirantis Y, Provata A (1999) Scaling properties of coding and non-coding DNA sequences. J Stat Phys 97: 233-262.

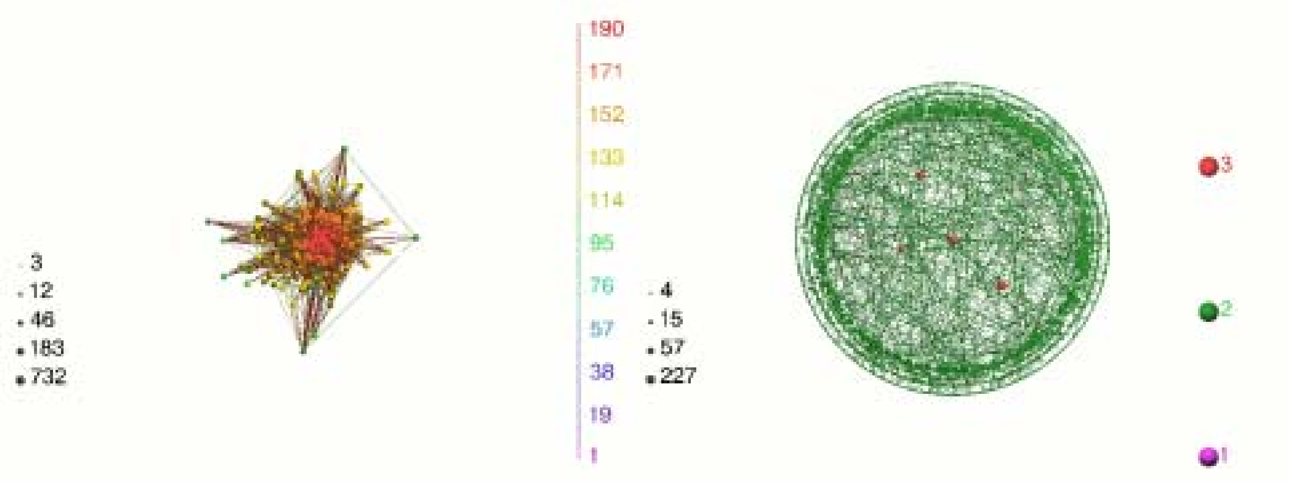

Figure 1: The k-core analysis of the content-based network of Balcan and Erzan * (left panel) and the Barabasi-Albert (BA) model **. In the left panel, the number of nodes is 745, and the number of edges 109247. The individual sequences obey the length distribution $p(l) \propto q^l$, with $q = 0.95$. The BA model network (right panel) has 5000 nodes, and is built by starting from a fully connected four-cluster and adding nodes with two edges at a time. The number of edges is 9998. In the k-core plot for the latter, only % 5 of the edges are shown for better visibility. The size of node is proportional to its degree, with the numbers corresponding to the different sizes being given to the left of each figure. The legend to the right of the figures shows the color coding of the different k-shells.

Table 1: The summary of our model ensemble with the power law distribution of PR lengths. The average number of interacting genes, regulatory genes (coding TFs), and interacting pairs obtained from one hundred realizations of our model ($\pm$ the standard deviations) with μ in the range $-0.5 \leq \mu \leq 2.5$.

μ	Genes	Regulatory	Interacting Pairs
-0.5	4962 ± 130	203 ± 14	21727 ± 2798
0.0	4290 ± 163	201 ± 14	15356 ± 1996
0.5	3554 ± 210	193 ± 15	10085 ± 1407
1.0	2864 ± 198	179 ± 13	6310 ± 862
1.5	2365 ± 220	168 ± 13	4218 ± 625
2.0	1985 ± 212	156 ± 12	2974 ± 452
2.5	1714 ± 206	130 ± 11	2285 ± 398

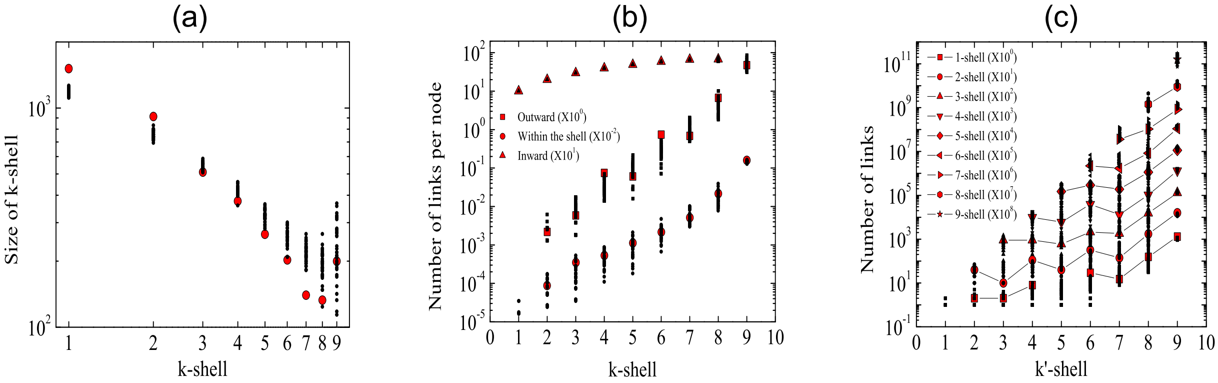

Figure 2: A quantitative comparison of k-core hierarchy of the networks obtained from the Yeastract data and the model. The red symbols pertain to Yeastract data whereas the black symbols represent model realizations. We have taken only the 36 realizations (out of 100) with $k_{\mathrm{max}} = 9$, for ease of comparison.
(a) k-shell populations. Note that in both the model and the experimental yeast network, the innermost core is over-represented with respect to the common trend, which is approximately proportional to k^{-1}.
(b) Average number of links per node that are radially outward (connecting to nodes within shells with $k' < k$), inward ($k' > k$), and within the shell ($k' = k$), as a function of the shell-number k. The labels "outward" and "inward" refer only to the circular arrangement of nodes placed at a greater or smaller distance from the center of the figure in the k-core visualization employed in Fig. 2 of the main text, where the directionality of the edges is ignored. Note that the different sets of model values and data points have been shifted with respect to each other for greater clarity, the "inward" connections upwards by one decade, and the same-shell connections, downwards by two decades.
(c) Distribution of the number of links connecting nodes in various k-shells (different symbols) with nodes in $k' \geq k$ shells. The different k-series have been offset with respect to each other for greater visibility, and the experimental points of each series connected as a guide to the eye.

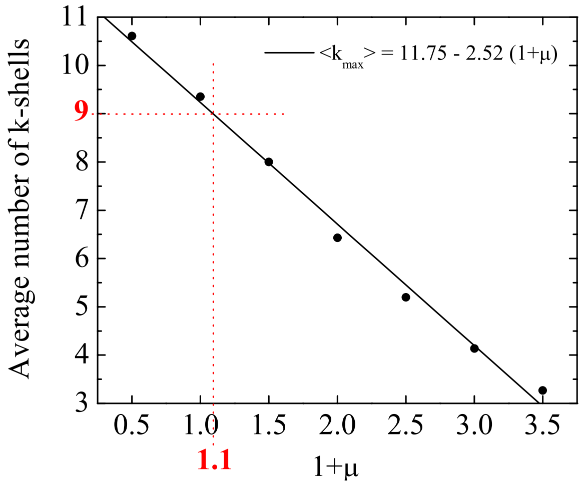

Figure 3: Average number of k-shells as a function of $1+\mu$, the exponent of the PR length distribution, $p_{\rm PR}(l) \sim l^{-1-\mu}$.

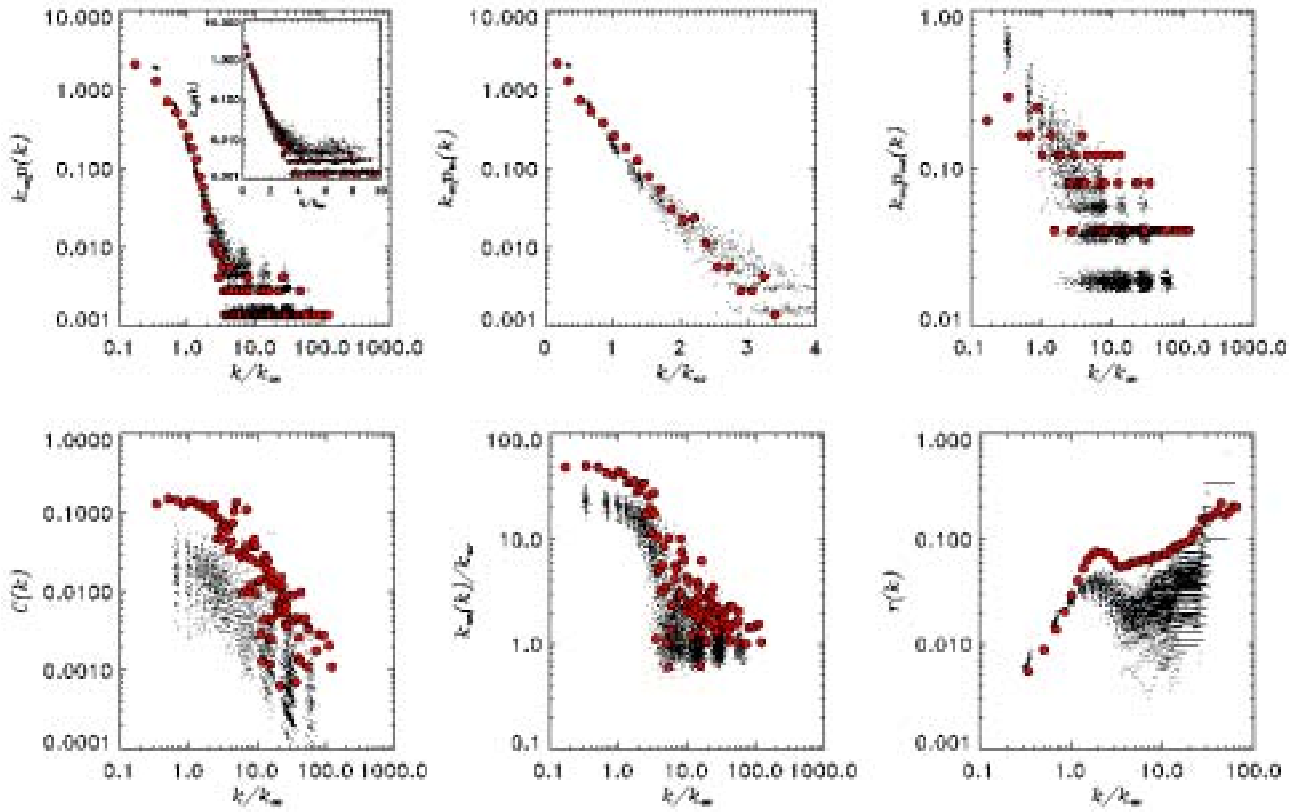

Figure 4: The topological features of the model network computed for $\mu = 2$. For larger values, the length distribution would cease to be fat tailed as suggested by Almirantis and Provata[††].

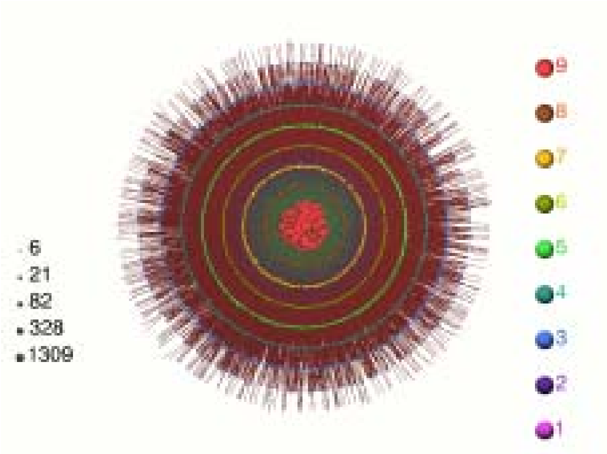

Figure 5: The k-core decomposition of one realization of the model network with the lengths of the PR sequences fixed at $L = 127$. The value of L was chosen to make $k_{\max} = 9$, to coincide with the corresponding value for the Yeastract data.

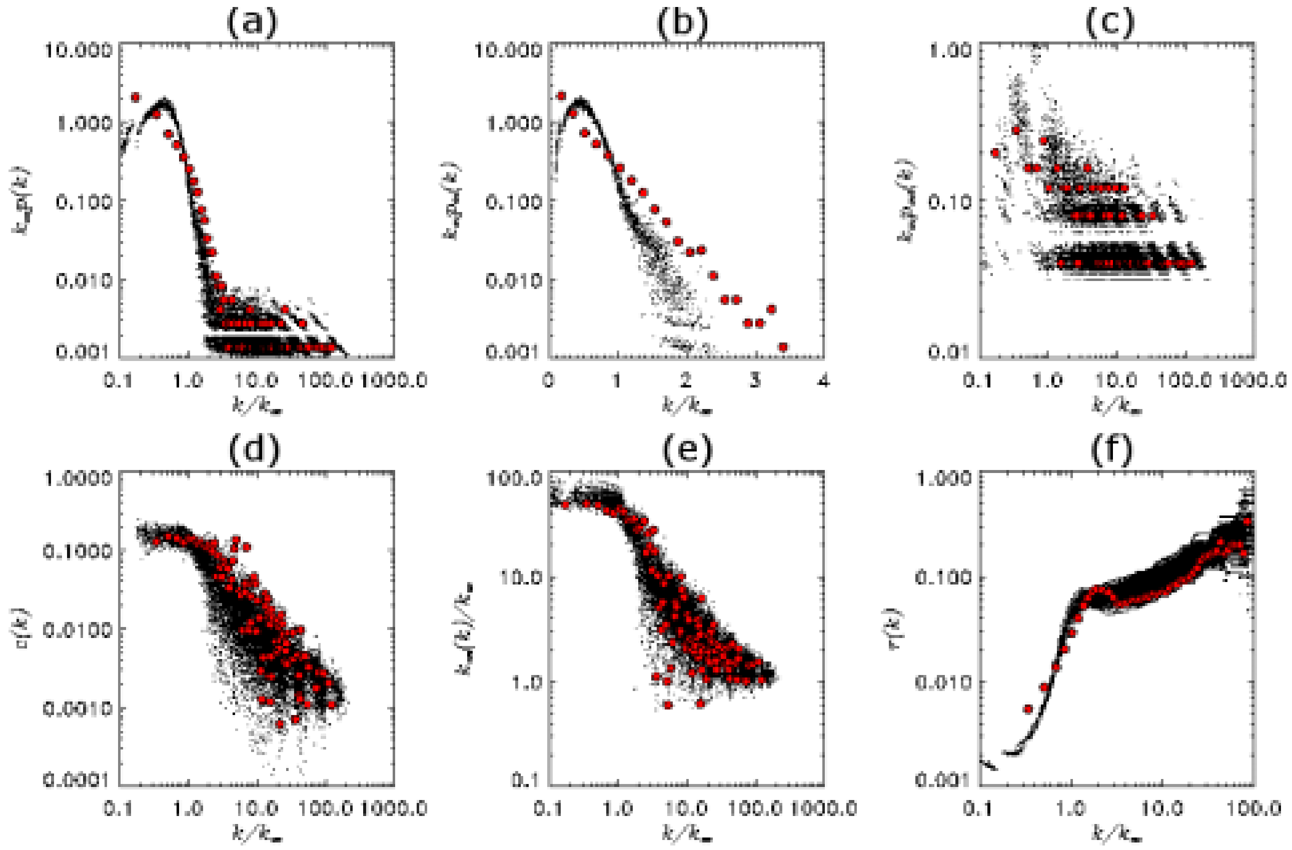

Figure 6: Superposition of Yeastract results and the scatter plots obtained from one hundred realizations of the model networks, with the lengths of the PR sequences fixed at $L = 127$. The value of L was chosen to make $k_{\max} = 9$. Although the plots look superficially like the ones reported in Fig. 3 of the main text, note, in particular, that the exponential decay of the in-degree distribution (and the resulting total degree distribution) have been modified, with a maximum around $k/k_{av} = 1$ and a faster than exponential decay. The rich-club coefficient has lost the distinctive shoulder.

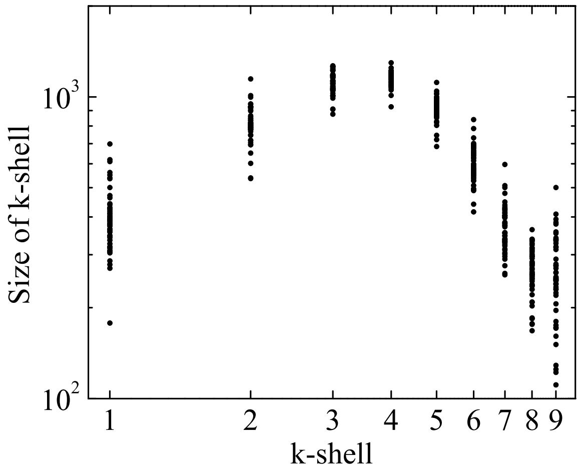

Figure 7: The distribution of nodes over different k-shells, for the null hypothesis for the lengths of the promoter regions, namely a fixed PR length of $L = 127$. At this value of the PR length, one finds a superficial similarity of the k-core visualization of this reduced model network with that of yeast. This figure should be contrasted with the distribution for the null-model shown in Fig. 2a.
