# Supplementary material for: The Information Coded in the Yeast Response Elements Accounts for Most of the Topological Properties of Its Transcriptional Regulation Network
Source: PLoS One. 2007 Jun 6;2(6):e501. doi: 10.1371/journal.pone.0000501 (PMC1876808; doi:10.1371/journal.pone.0000501)
Supplement: Text S3 — Ranking of Overlapping Sets of Regulated Genes and Sequence Inclusion (0.02 MB PDF) [file pone.0000501.s003.pdf]

Text S3
Ranking of Overlapping Sets of Regulated Genes and Sequence Inclusion

Supporting Information for
The Information Coded in the Yeast Response Elements Accounts for most of the Topological Properties of its Transcriptional Regulation Network
Duygu Balcan, Alkan Kabakçıoğlu, Muhittin Mungan, Ayşe Erzan

We here report a statistical fact in support of the basic assumption underlying our model. The matching condition we employ dictates a certain correlation between the sets of regulated genes by each TF: if the binding sequence of a TF (A) is embedded in that of a TF (B), then the set of genes $\{G_i\}_B$ regulated by TF_B in our model is a subset of $\{G_i\}_A$. A similar investigation of the yeast databases listed below reveals that the top 50% of the TF pairs related by the sequence inclusion relation above, rank in the top 3% when all the TF pairs are listed according to the overlap of their $\{G_i\}$ sets. The actual ranking of the TF pairs obtained among all possible pairs of 102 TFs with known binding sequences is shown in Fig. 1.

On the other hand, the more straightforward expectation that TFs with short binding sequences should regulate more genes is not verified by the same data. This curious fact probably points to certain sequence correlations arising from the duplication and divergence processes [†] that distort the occurrence statistics of the binding sequences in PRs. Note that the result in Fig. 1 is robust to such deviations from the unbiased probabilities for the occurrence of different strings.

[†] Wagner A (1994) Evolution of gene networks by gene duplications: a mathematical model and its implications on genome organization. Proc Natl Acad Sci USA 91: 4387-4391.

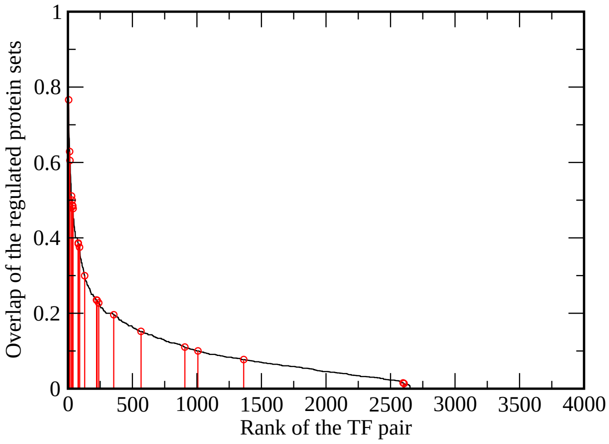

Figure 1: Correlation between the sets of proteins regulated by the TFs with similar binding sequences. The vertical axis is the percentage overlap of the two sets of genes regulated by an arbitrary pair of TFs, which are ranked on the horizontal axis according to their overlap. The red vertical lines mark those pairs of TFs that are also related by binding sequence inclusion. The accumulation of the red lines to the left of the graph is indicative of the correlation described in the text.
