# Supplementary material for: The Information Coded in the Yeast Response Elements Accounts for Most of the Topological Properties of Its Transcriptional Regulation Network
Source: PLoS One. 2007 Jun 6;2(6):e501. doi: 10.1371/journal.pone.0000501 (PMC1876808; doi:10.1371/journal.pone.0000501)
Supplement: Text S4 — Comparison of the Model with the Yeast Data from Different Databases (0.04 MB PDF) [file pone.0000501.s004.pdf]

Text S4

Comparison of the Model with the Yeast Data from Different Databases

Supporting Information for

The Information Coded in the Yeast Response Elements Accounts for most of the Topological Properties of its Transcriptional Regulation Network

Duygu Balcan, Alkan Kabakçıoğlu, Muhittin Mungan, Ayşe Erzan

We display in Fig. 1, for comparison, the topological coefficients as computed from different data sources for the yeast TRN, superposed on the scatter plots for the ensemble of model networks. We see that although there are partial differences between the data sets, all are compatible with the model results. Since the different data sets encompass slightly different sets of genes, this goes further to show that the model captures the essential building principles of the networks.

MacIsaac et al.[†] have recently proposed an improved gene map for regulatory sites of *Saccharomyces cerevisiae*. The supplementary data associated with their work, however, contains a more restricted set, namely the conserved gene regulatory interactions common to at least three yeast species. It is nevertheless interesting to note that the interaction network obtained under this restriction display topological properties similar to those shown in Fig. 1.

[†] MacIsaac K, Wang T, Gordon DB, Gifford DK, Stormo GD, et al. (2006) An improved map of conserved regulatory sites for *Saccharomyces cerevisiae*. BMC Informatics 7: 113-127.

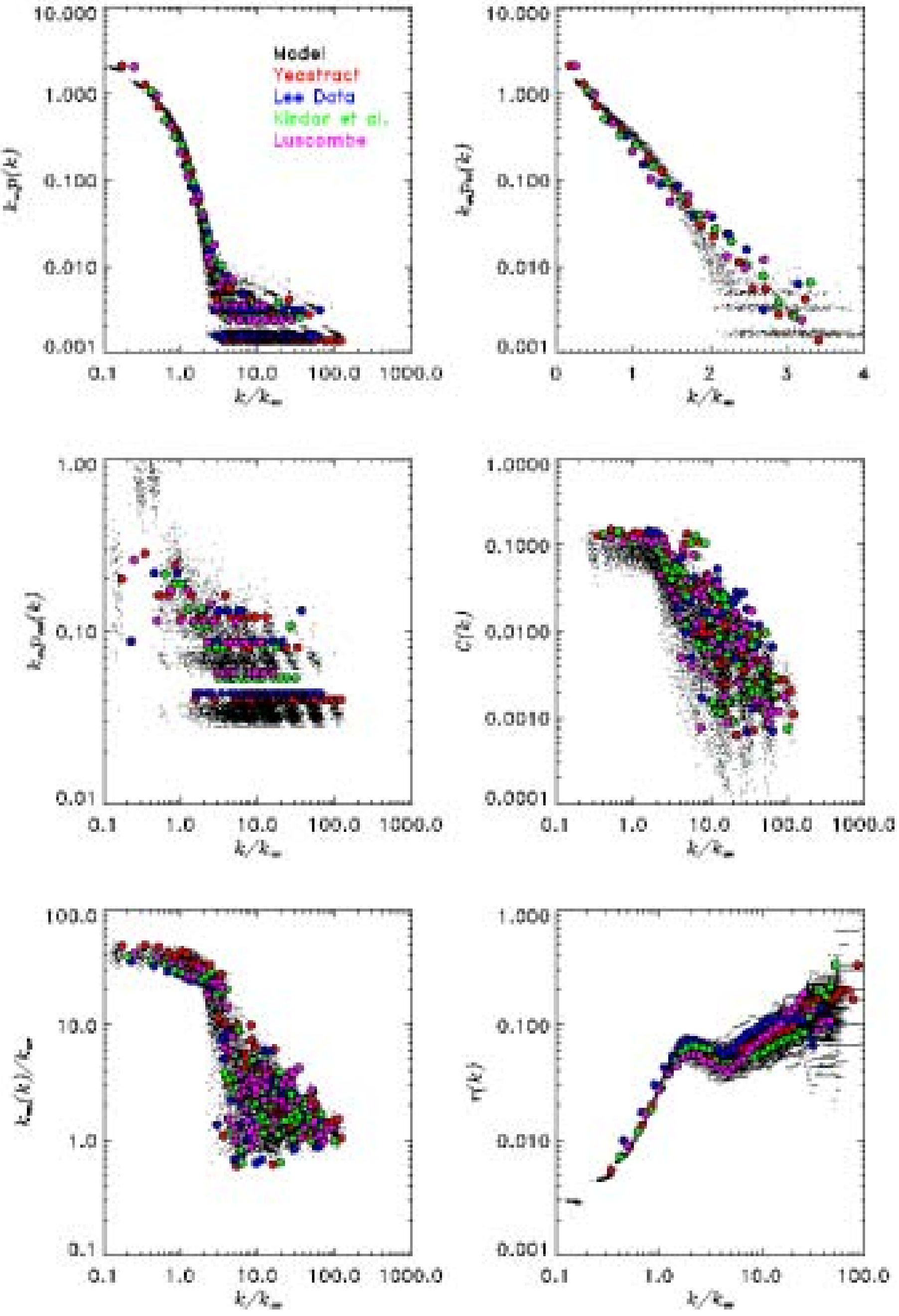

Figure 1: The network statistics extracted from the sources listed in Table 1 in main text superposed on the simulation results corresponding to one hundred realizations of the model network (black dots). The agreement is extremely good with all of these sets of data, which almost completely cover, but do not exceed the phase space of our model. (Black, red, blue, green and magenta correspond to the model, Yeastract, Fraenkel Lab, Kırdar and Luscombe data respectively. See Table 1, main text.)
