# Supplementary material for: The Information Coded in the Yeast Response Elements Accounts for Most of the Topological Properties of Its Transcriptional Regulation Network
Source: PLoS One. 2007 Jun 6;2(6):e501. doi: 10.1371/journal.pone.0000501 (PMC1876808; doi:10.1371/journal.pone.0000501)
Supplement: Text S5 — Randomization Procedures and Null-Null-Models (2.19 MB PDF) [file pone.0000501.s005.pdf]

Text S5
Randomization Procedures and Null-Null-Models

Supporting Information for
The Information Coded in the Yeast Response Elements Accounts for most of the Topological Properties of its Transcriptional Regulation Network
Duygu Balcan, Alkan Kabakçıoğlu, Muhittin Mungan, Ayşe Erzan

In this supplement we will compare our null-model with randomized versions thereof, as well as with a configurational model, an Erdös-Rényi type model and a hidden-variable model.

Randomizing the edges of the model network and the yeast network. We compared the k-core decompositions, clustering coefficients, the degree-degree correlations, the rich-club coefficients of the Yeastract data with those obtained after randomly reconnecting the edges of the network while *i)* keeping the in- and out-degree of each node fixed separately and *ii)* only keeping the degree of each node fixed, irrespective of the directionality.

In Fig. 1a we display the *randomized* k-core plots for yeast and the same realization of the model network whose k-core plot was displayed in Fig. 2 of the main text. We randomly choose pairs of edges and exchange their terminals, either the in with in, or the out with out. We repeat this procedure $2 \times E$ times, where E is the total number of edges. This preserves the directionality of the edges, as well as the in- and out-degree of each node separately.

In Fig. 1b we show parallel results obtained when we ignore the directionality of the edges under the random rewiring procedure, but conserve the total degree of each node.

In all of the above, bidirectional connections were counted as two different edges, and thus this procedure does not conserve bidirectionality. However, these connections comprise less than 1% of the total, and can be ignored altogether.

We see that the randomization procedure which preserves directionality essentially leaves the k-core structure invariant, while randomizing without respecting directionality of the edges produces a strikingly different picture, both for the yeast and the model network; $k_{\max}$ becomes 29±1 rather than 9. While in the yeast and model networks, the largest fraction of connections is to the inner-

most shell, the $k_{\max}$-core (see Fig. 2 of the main text and Fig. 2 of Text S2), in the randomized networks without direction conservation, there is a high degree of intra-shell connectivity. The topological coefficients are also completely altered in this case, as displayed in Fig. 2.

The configuration model. An ensemble of networks generated from one model realization by randomizing the edges while keeping the directionality fixed, as is done above, is in fact equivalent to a configuration model†. The configuration model is obtained by taking the in- and out-degree sequence of one realization of the content-based network, removing the edges, exchanging the in- with the out-degree assignments between randomly chosen pairs of nodes to remove any possible residual correlations between these quantities, attaching corresponding numbers of arrows to the nodes, and then randomly connecting pairs of in and out arrows. The in- and out-degree distributions are statistically identical to the content-based model, and the rest of the topological features are indistinguishable from each other, to the extent that they are determined by the in- and out-degree distributions. The results agree very closely with the topological properties of the networks as computed from Yeastract data (see Fig. 3 of the main text, and the top row of Fig. 2). The crucial fact to keep in mind is that the in- and out-degree distributions of our model are *not* imported from any data set, but independently generated by the information sharing mechanism embodied in the string-matching condition underlying our model, given the effective length distributions for the TFs and the PRs extracted from the yeast data.

A modified Erdös-Rényi model. An Erdös-Rényi type null-null-model can be obtained by picking a subset (of size $N_{\rm TF}$) of all the nodes (N), and allowing only these (i.e., TF-coding) nodes to have out-edges to randomly picked nodes in the whole network, with a probability p. This probability is to be fixed by the density of edges on the real yeast TRN, i.e., $p = E/E_{max}$, where E is the number of edges, and $E_{max} = NN_{\rm TF}$ the total number of possible edges in this network. The resulting network (see Fig. 3) has a bi-modal degree distribution consisting of the superposition of two well-separated Poissonian peaks, centered over the mean in- and out-degree contributions, E/N and $E/N_{\rm TF}$. The topological features are qualitatively different from our null-model and the yeast TRN in all respects. In particular, the bi-modal degree distribution gets reflected in disconnected plots of degree-degree correlations and the clustering coefficient, with essentially degree-independent small-degree regions (coming from the relatively small in-degrees) and a disconnected large degree part coming from the out-degrees of the TF-coding nodes. The k-core decomposition, on the other hand, indicates a highly hierarchical structure and looks indistinguishable from Fig. 2 of the main text, with $k_{\max} = 7$. A closer inspection reveals, however, that the distribution of the number of

nodes over the different shells is in fact qualitatively different, resembling that found when the lengths of all the PR sequences are set to a large number well separated from the TF lengths. (see Text S2).

Comparison with a hidden-variable model. A mean-field version of our present null-model resembles the hidden-variable model of Caldarelli et al. [***]. A mean field version of our model can be constructed by assigning to each node i, two random variables l_i, k_i, distributed in the same way as the lengths of the TF and the PR strings associated with the organism under consideration. (To account for the fact that only a fraction $\eta = 4.8\%$ of the nodes code for TFs, in practice, $1-\eta$ of the nodes are assigned "TF lengths" exceeding the maximum "PR lengths.") These hidden-variables take the place of the TF and PR strings associated with the nodes. To simulate this effective model, we use the *ensemble averaged probabilities* $p(l_i, k_j)$ and $p(l_j, k_i)$ derived from the string matching condition[+], for inserting directed edges between the nodes (i, j) (see Eq. 1, main text).

We show in Fig. 4 the simulation results for the topological features of the hidden-variable model, averaged over 100 realizations and superposed on the ensemble averages of our content-based model. For each realization we start with 6000 nodes; around 2000 nodes have zero total degree, and there also exist few two-clusters (the nodes thus having unit degree). The rest belong to the giant cluster. Since the model features were computed only over the giant connected cluster, here the statistics are performed also over the giant connected cluster in each realization.

The ensemble averaged results of the hidden-variable model and the content-based model are very close to each other, except that the content-based model has an in-degree distribution with a longer tail (in the range $15 \leq k \leq 32$) than the hidden-variable model, albeit with very small probabilities. This gives rise to small differences in the other topological features in this degree range.

The subtle difference in the rare event range of the in-degree distribution may arise from an underestimation of the connection probabilities $p(l, k)$ by the mean field approximation[+].

[†] Molloy M, Reed B (1995) A critical point for random graphs with a given degree sequence. Random Structures and Algorithms 6: 161-180.
[***] Caldarelli G, Capocci A, De Los Rios P, Munoz MA (2002) Scale-free networks from varying vertex intrinsic fitness. Phys Rev Lett 89: 258702.
[+] Mungan M, Kabakcioglu A, Balcan D, Erzan A (2005) Analytical solution of a stochastic

content-based network model. J Phys A 38 (44): 9599-9620.

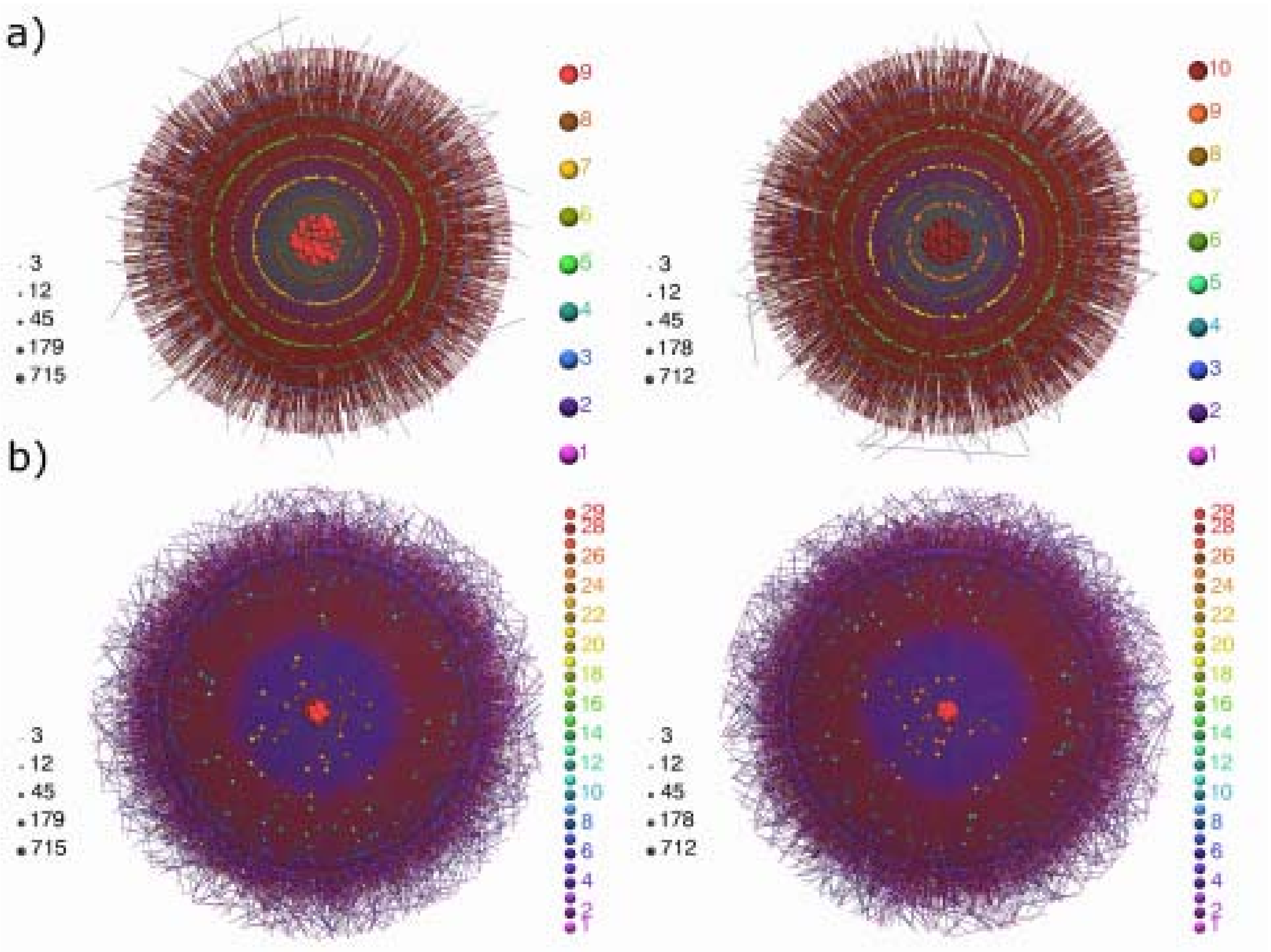

Figure 1: The k-core analysis of the randomized versions of the model (left panel) and Yeastract (right panel) networks, preserving separately the in- and out-degrees (a) or the total degree (b) of each node. The first set of k-core plots (a) are essentially indistinguishable from Fig. 2 of the main text. In contrast, those obtained by the randomization procedure ignoring the directionality of the edges (b) are strikingly different. The number of shells have gone up to 29 from 9, and the much higher intra-shell rather than inter-shell connectivity (as can be seen by following the edges) indicates that the hierarchical nature of the yeast network, which is faithfully reproduced by the model, is destroyed by the nondirectional randomization process.

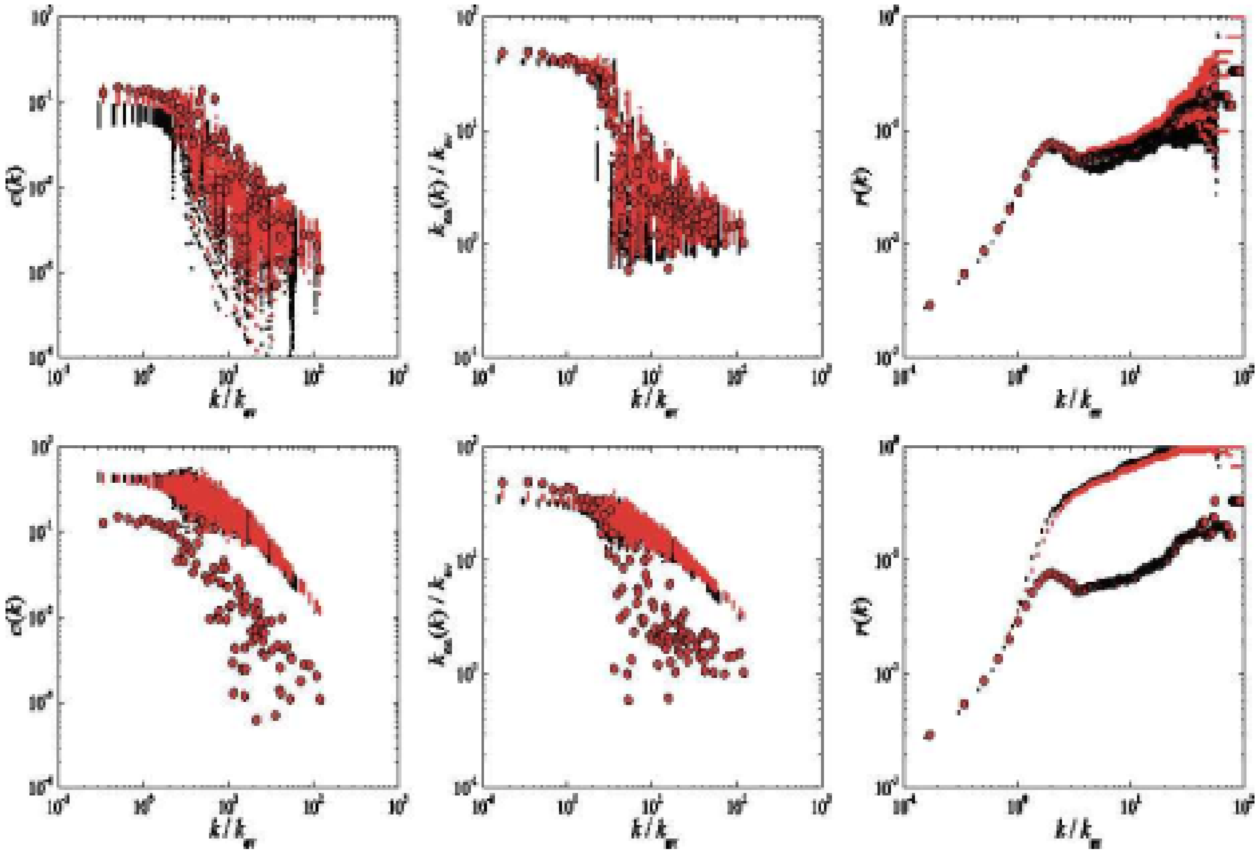

Figure 2: The effect of the same randomization procedure in as in Fig. 1 on the clustering coefficient (left), the degree-degree correlations between neighboring nodes (center), and the rich-club coefficient (right) of the Yeastract data (red circles) and one realization of the model network. In the top row, we show the results of keeping in- and out-degrees of each node fixed, while the bottom row only preserves the total degree of each node. The black dots correspond to an ensemble of one hundred independent randomizations of one realization of the model network and the red dots to a similar set of randomized versions of the Yeast network.

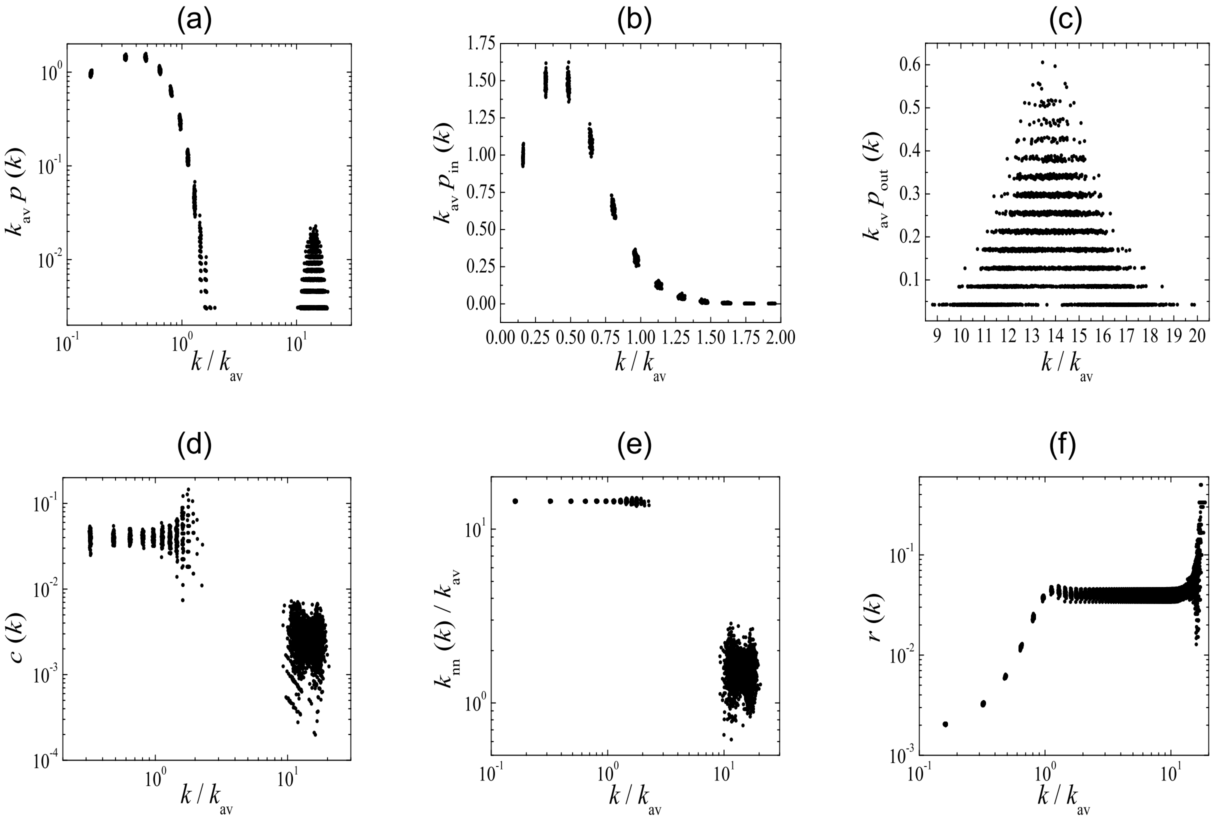

Figure 3: The topological features of the Erdös-Rényi random network version of the TRN network, with the connection probability determined only from the density of edges of the empirical network (Yeastract data).

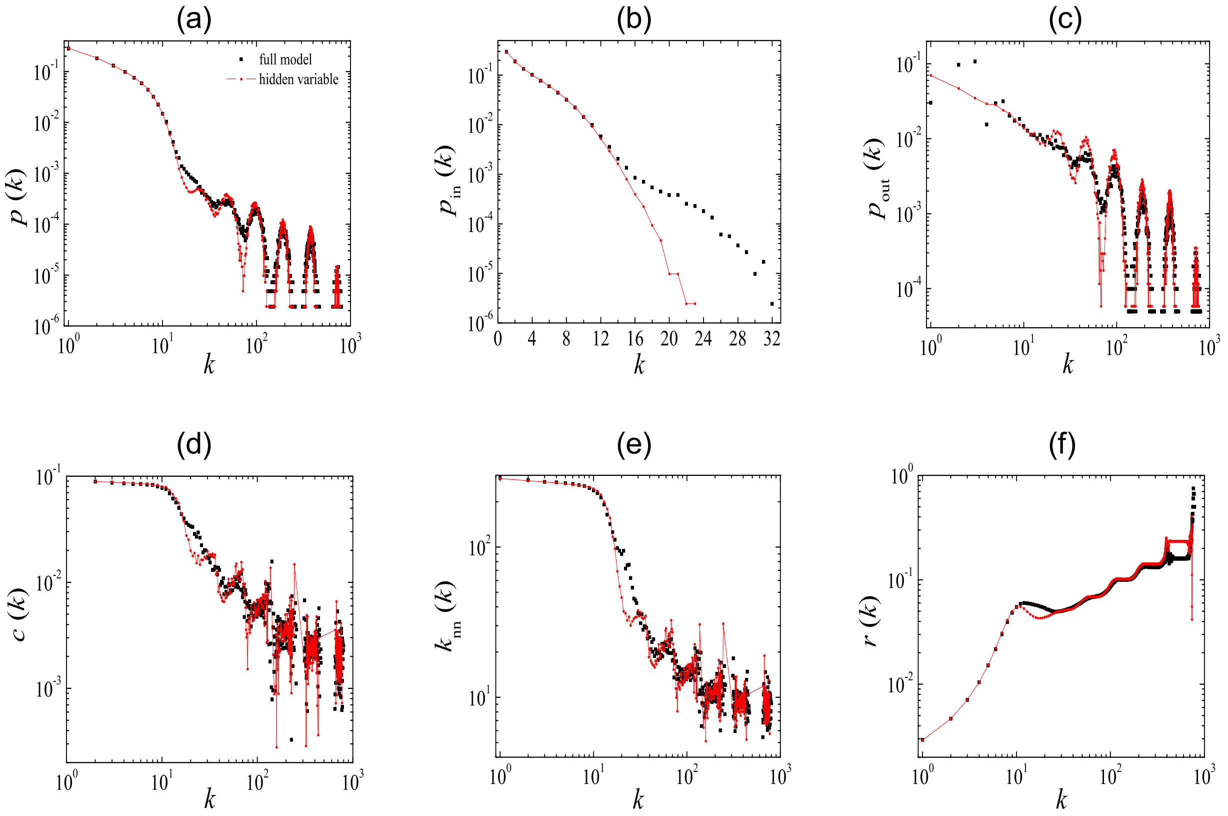

Figure 4: The topological features of the hidden-variable model, with $N = 6000$ nodes, ensemble averaged over 100 realizations, superposed on the ensemble averaged results for the null-model. Only the giant connected component is shown. See text.
